# Visible Light Induces Melanogenesis in Human Skin through a Photoadaptive Response

**DOI:** 10.1371/journal.pone.0130949

**Published:** 2015-06-29

**Authors:** Manpreet Randhawa, InSeok Seo, Frank Liebel, Michael D. Southall, Nikiforos Kollias, Eduardo Ruvolo

**Affiliations:** Johnson and Johnson Skin Research Center, CPPW, a unit of Johnson & Johnson Consumer Companies Inc., Skillman, New Jersey, United States of America; San Gallicano Dermatologic Institute, ITALY

## Abstract

Visible light (400–700 nm) lies outside of the spectral range of what photobiologists define as deleterious radiation and as a result few studies have studied the effects of visible light range of wavelengths on skin. This oversight is important considering that during outdoors activities skin is exposed to the full solar spectrum, including visible light, and to multiple exposures at different times and doses. Although the contribution of the UV component of sunlight to skin damage has been established, few studies have examined the effects of non-UV solar radiation on skin physiology in terms of inflammation, and limited information is available regarding the role of visible light on pigmentation. The purpose of this study was to determine the effect of visible light on the pro-pigmentation pathways and melanin formation in skin. Exposure to visible light in *ex-vivo* and *clinical* studies demonstrated an induction of pigmentation in skin by visible light. Results showed that a single exposure to visible light induced very little pigmentation whereas multiple exposures with visible light resulted in darker and sustained pigmentation. These findings have potential implications on the management of photo-aggravated pigmentary disorders, the proper use of sunscreens, and the treatment of depigmented lesions.

## Introduction

Visible light (VL) is characterized by wavelengths ranging from 400 to 700 nm, although in some documents of the European Commission it has been defined to cover the range of 380–780 nm CIE [1]. In this paper VL is defined as covering 400–700 nm. The VL part of the spectrum has not been thoroughly investigated as compared to UV radiation, regarding both positive and side effects [2]. VL has been reported to induce both transient [3] as well as long lasting pigmentation in human skin [4]. It was shown that VL induced pigmentation may persist up to 8 weeks and the amount of pigment produced is dependent on the total dose of light. A recent investigation into the effects of VL in human skin especially in people with darker complexion [5] showed that the induction of pigmentation was not accompanied by any deleterious effects such as those induced by ultraviolet (UV) radiation. In contrast to these observations, Liebel F. et al [6] showed that VL can induce significant ROS production resulting in release of pro-inflammatory cytokines and matrix metalloproteinases (MMPs) expression in skin.

Skin plays a role as biological active barrier to the external environment including sun exposure and the presence of cutaneous hyptothalamic-pituitary-adrenal axis (HPA) makes it an important peripheral neuroendocrine organ [7]. The skin acts not only as a target for neuroendocrine signals but also a source of hormones and neurotransmitters, particularly the epidermis [8]. As a result biological responses for pigmentation formation do not necessarily share the same mechanism of action to environmental insults including different wavelengths of sun exposure. A single exposure of UVB can induce delayed pigment formation preceded by an erythema response. UVA (320–400 nm) can induce IPD (immediate pigment darkening) during the first minutes of exposure, which is transient form of pigmentation and fades away within few hours [9, 10], or PPD (persistent pigment darkening) that appears within hours of higher doses of UVA exposure and persist up to several days or weeks [11, 12]. Both IPD and PPD as well as erythema has been shown to be induced in skin phototypes I and II by single UVA exposure [13] and some studies in fair skinned persons have also investigated the increase in pigmentation after multiple exposures of UVA [14–21]. Traditionally skin pigmentation is believed as the most important photoprotective factor, since melanin, besides functioning as a broadband UV absorbent, has antioxidant and radical scavenging properties. However UVB induced melanin provides coverage against subsequent UV damage via increased melanin production supplemented by the redistribution of melanin towards the upper layers of the skin, whereas UVA induced tanning which is as result of photooxidation of existing melanin substrate provides very little coverage in the way of photoprotection [22]. Although UVB and UVA portions of solar spectra are very well studied, but there is a lack of published studies on the effect of VL of pigment induction in skin.

In this paper a series of *ex-vivo* and *clinical* studies were performed to understand the induction of pigmentation in skin with VL. In the first series we explored the induction of pigment in human skin explants extracted from abdominal skin in response to multiple exposures with VL. Melanin deposition as a result to exposure with VL was more evident at later time points. Further investigation of melanogenesis pathway in *ex-vivo* showed de-novo formation of melanin. In the second series we were able to reproduce the results obtained from explants study and we demonstrated clinically perceptible pigmentation after multiple exposures with VL. A single exposure to VL might prove ineffective in producing persistent pigment; therefore a pre-exposure of Caucasian skin to what might be termed, as “conditioning” dose has proved quite effective in augmenting the pigment resulting in clinically perceptible pigmentation.

## Results

### Exposure to VL results in increased melanin formation in skin explants

**Fig 1:**
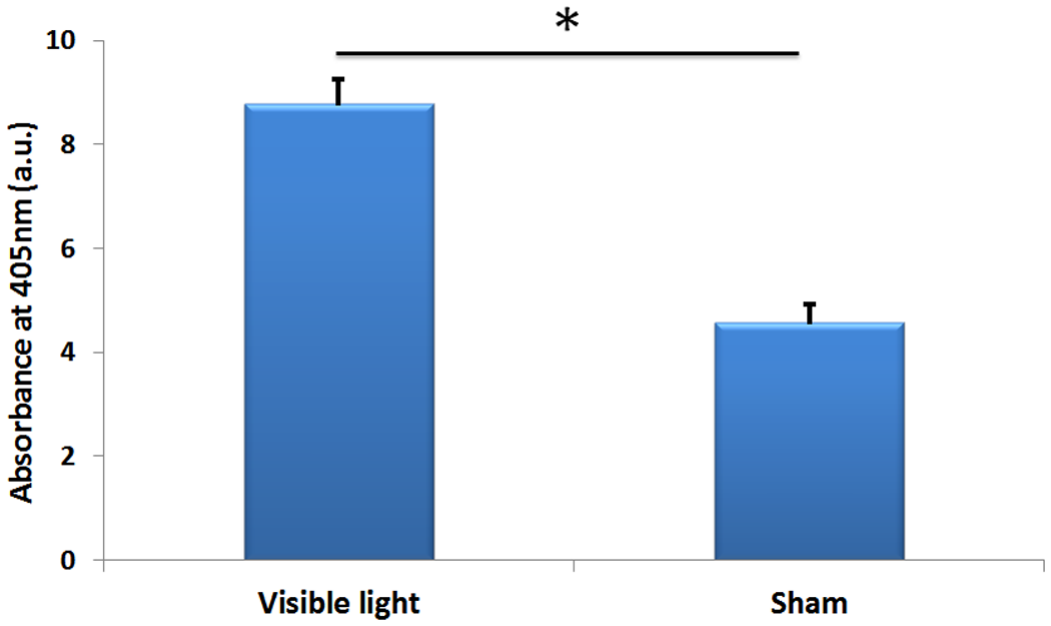
Visible Light Enhanced Pigment Deposition in human skin explants. Human skin explants were exposed to visible light 150 J.cm-2 (day 1, 2, 3, 6, 7) and harvested at day 7 for quantification of melanin content.

Skin explants exposed to 150 J.cm^-2^ of VL demonstrated an increased darkening as a function of exposures. VL exposed explants at 7 days documented increased melanin content as compared to sham treated samples (Fig 1).

**Fig 2:**
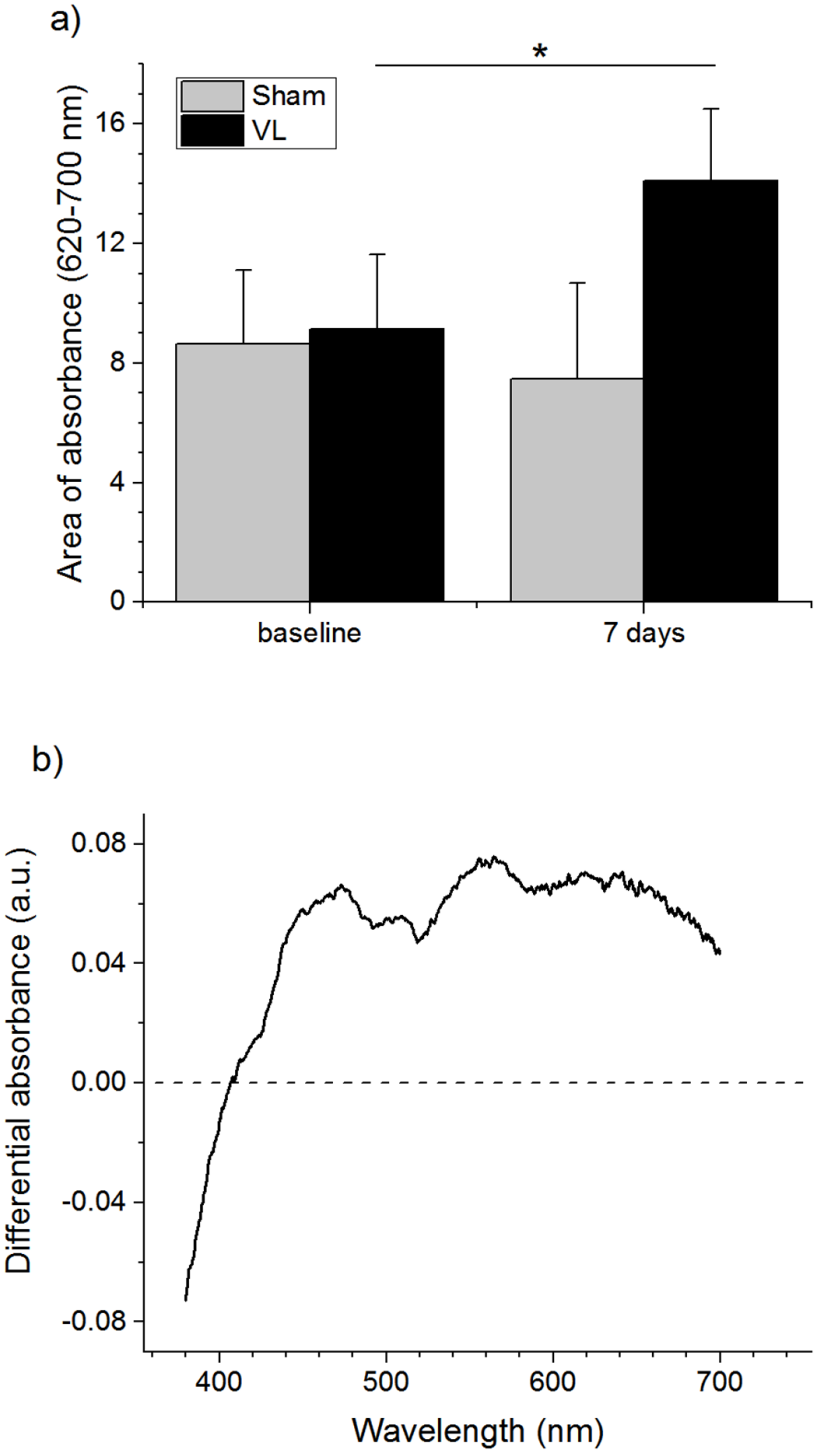
DRS results showing the changes of absorption induced by visible light exposures on the skin explants. A) Pigmentation represented by the area of differential absorption at 620–700 nm. Only the samples of visible light exposure showed absorption changes with statistical significance (p < 0.05). B) Representative spectral changes in absorption between baseline and post-exposure on day 7.

The amount of pigment induced by VL exposure on skin explants was also assessed by diffuse reflectance spectroscopy (DRS). DRS absorption levels obtained at wavelengths from 620–700 nm is shown in Fig 2A for sham and VL exposed explants. A significant absorption change (*p* < 0.05) was observed on day 7 for the exposed samples compared to baseline, whereas there was no significant changes for the sham treatments. The changes of absorption in the whole visible spectral range between baseline and day 7 is presented in Fig 2B for the explants exposed to the VL. The spectral changes are very similar to what we observed from *in vivo* measurements [23].

### Exposure to VL alters pigmentation related gene expression in skin explants

**Fig 3:**
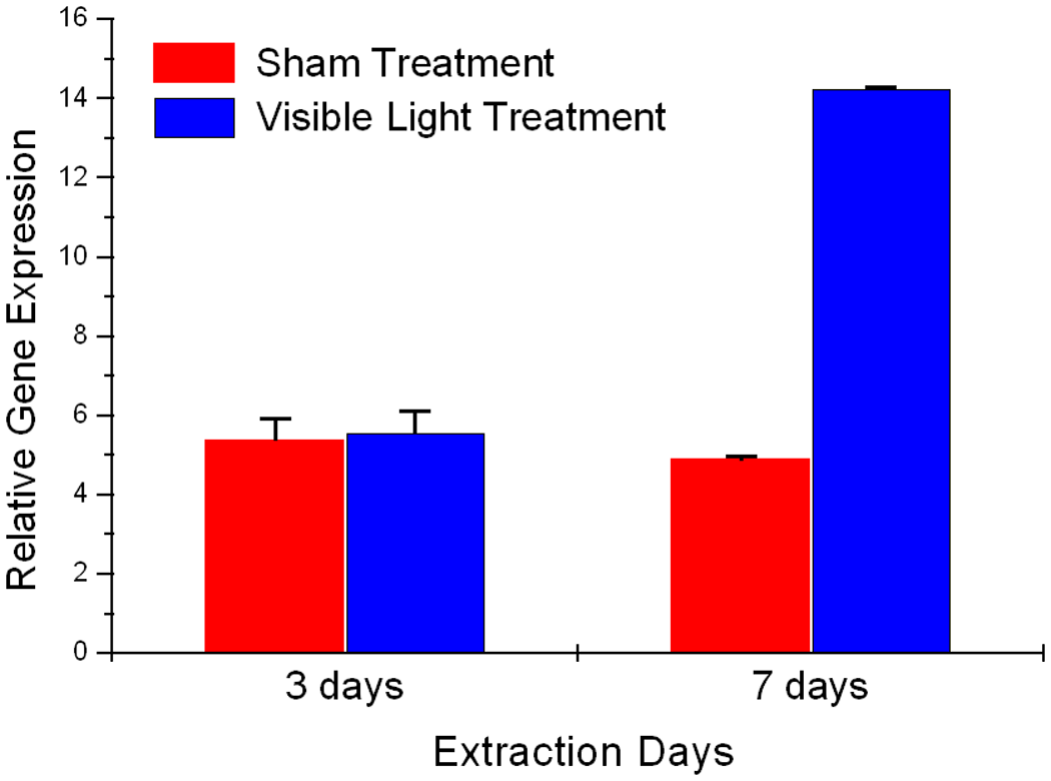
Relative expression of Tyrosinase gene expression in Visible Light-Exposed Skin Explants. Human abdominal skin biopsies were exposed to Visible light (150 J.cm-2 at day 1, 2, 3, 6, 7). Graph illustrates the relative expression in explants harvested day 3 and day 7 respectively.

The explants were analyzed for gene assays at day 3 and day 7 after exposure with sham and VL treatments respectively. The respective explants were analyzed for Tyrosinase gene expression by QPCR. A significant increase in Tyrosinase gene expression by almost 3 fold was registered on day 7 as compared with the sham exposure (Fig 3); however, the expression level remained the same for Tyrosinase gene expression at day 3. Also it should be noted that the expression level of Tyrosinase remained the same in sham-treated explants both on day 3 and 7 (Fig 3).

### Exposure to VL increases Tyrosinase enzyme activity

**Fig 4:**
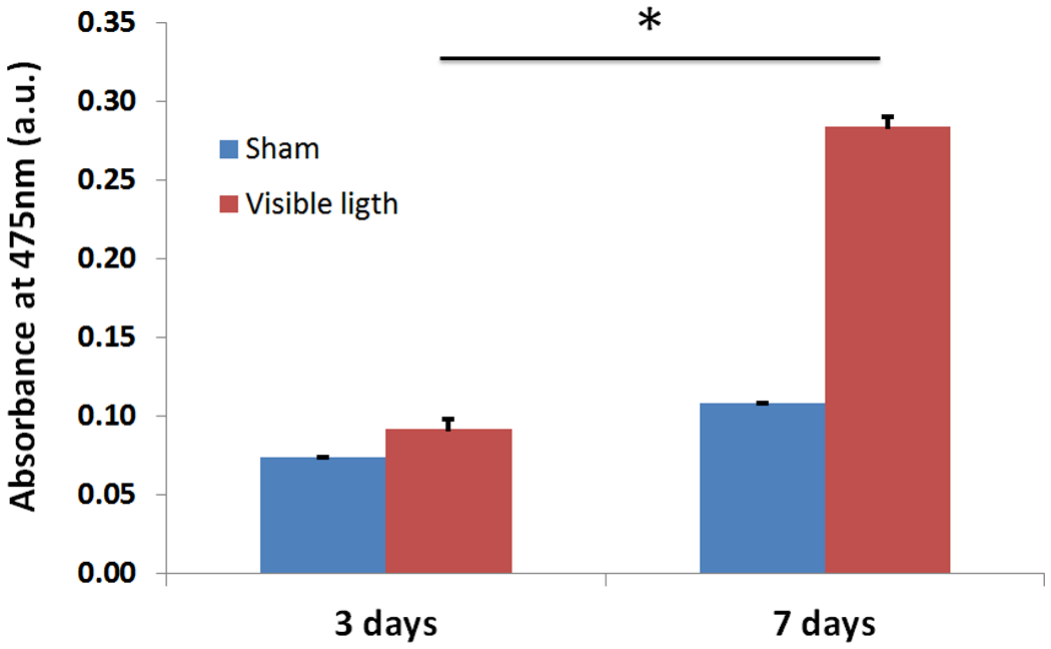
Tyrosinase enzyme activity at day 3 and day 7 for visible light exposure and sham treatment.

Tyrosinase activity, as the dopa oxidase here, was measured by the rate of L-DOPA oxidation as reported [24]. Tyrosinase enzyme activity in this study was evaluated in explants harvested at day 3 and day 7 after their respective exposure to VL. Dopachrome production was measured at 475 nm. Dopachrome formation during initial phase of oxidation of L-dopa by Tyrosinase enzyme present in the total protein content extracted from respective treatments at day 3 and 7 respectively shows higher Tyrosinase enzyme activity from VL exposed on both days as compared with sham treated samples (Fig 4). In addition increased enzyme activity was registered in VL treated explants on day 7 as compared to day 3 respectively, indicating that continuous exposure to VL can result in an increased Tyrosinase enzyme activity over the course of 7 days of treatment.

### Multiple exposures to VL induced pigmentation *in vivo*.

A pilot study involving individuals of Fitzpatrick skin type V-VI exposed to a single exposure of VL was conducted and the results, showed a transient pigment but no persistent pigmentation (subjects irradiated with 320 J.cm^-2^, data not shown). Of note, the single exposure study was conducted during beginning of December in the Northeastern United States, and thus occurred when subjects are not routinely exposed to the sun. A subsequent study was designed to determine whether multiple exposures to VL could induce persistent pigmentation and was based on the hypothesis that a “conditioning” dose of VL might affect the amount of pigment produced in skin.

**Fig 5:**
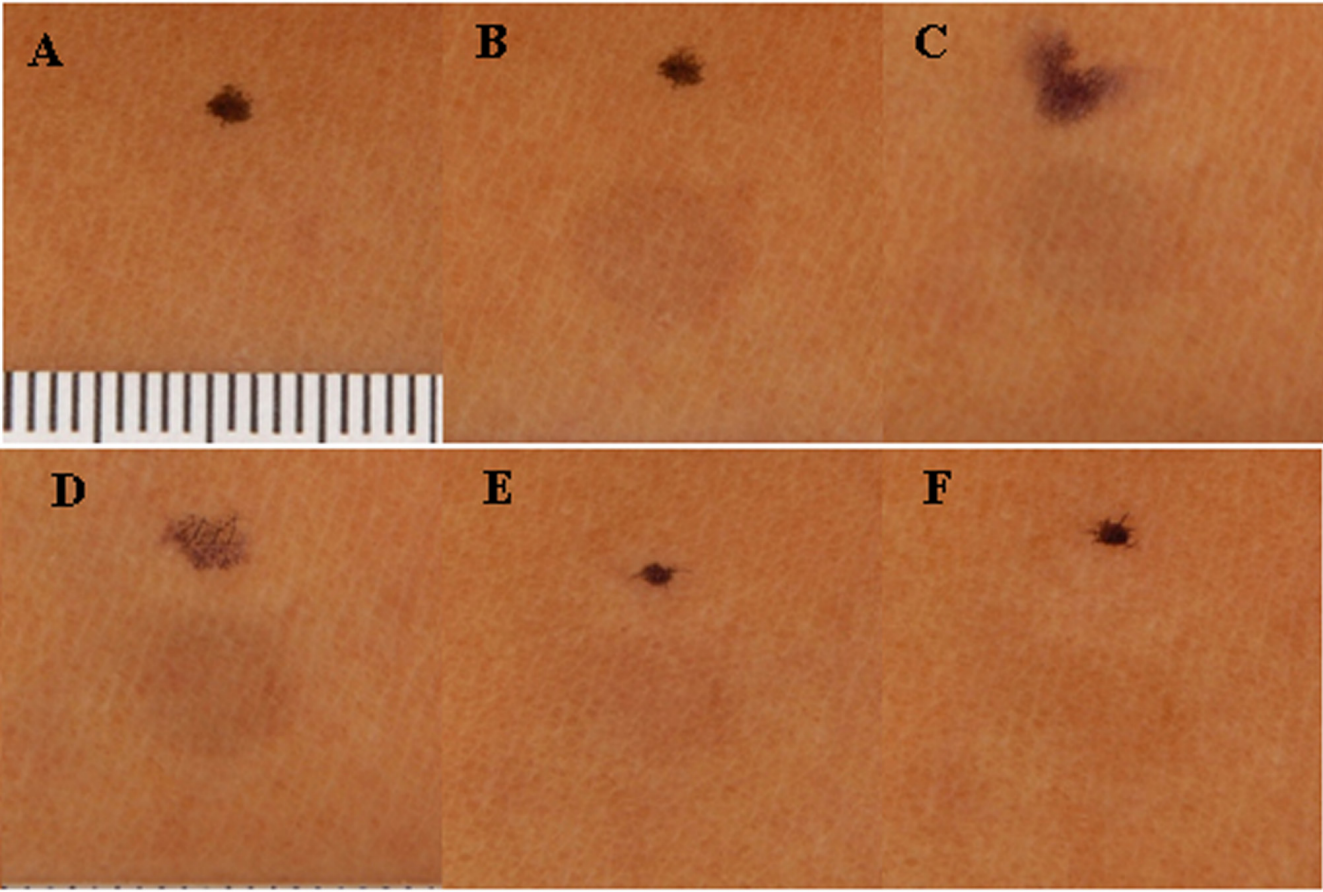
Illustrates the cross-polarized image of a subject skin phototype V for: a) baseline, no irradiation; b) 300 J.cm-2 day 1; c) 150 J.cm-2 day 2; d) 150 J.cm-2 day 3; e) no irradiation day 4; f) no irradiation day 10. The dots represent the permanent marker ink used to localized the irradiate site. The white scale bar is from the close up accessory that illustrates the millimeter scale to provide dimensional information. Space in between two consecutive black lines in the white scale represents 1mm distance.

Pigment was induced in the skin of subjects following an exposure to VL (300 J.cm^-2^) (Fig 5B). By 24 hours after the first VL exposure, the pigmentation induced on day 1 had mostly faded. The skin was exposed to a second exposure of 150 J.cm^-2^ VL and immediately following the second exposure pigmentation was increased to the same clinical grade as at the end of the first irradiation (Fig 5C) dose. Subsequent dose continued (Fig 5D) to increase pigmentation and the clinically perceptible pigmentation persisted for 10 days (Fig 5E and 5F). Similar results were clinically observed in all skin types.

**Fig 6:**
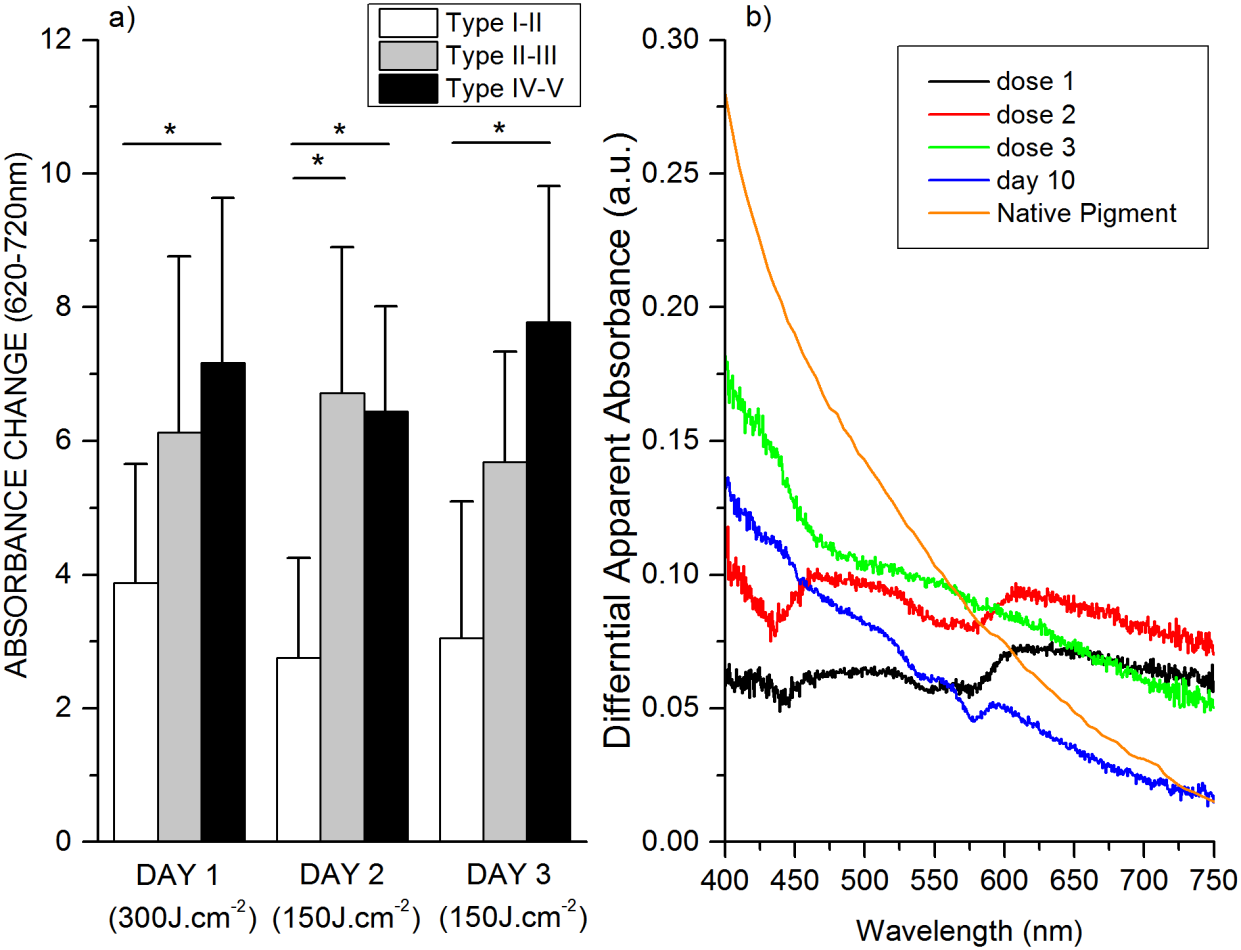
A) Illustrates the amount of facultative pigment induced after the initial conditioning dose of 300 J.cm-2 and the lower daily irradiation dose of 150 J/cm2 for different skin phototypes. Figure shows that a small daily dose increases or maintains the skin pigmentation induced by the initial conditioning dose. B) Spectral characteristics of the pigment content induced by visible light after multiple exposures. Spectral changes illustrate gradual changes from IPD type of response (day1) to a spectral feature that is similar to native pigment (day 10). For comparison, spectrum for native pigment is inserted to show gradual changes of pigmentation from multiple exposures of visible light to native pigment on day 10.

The pigmentation of the irradiated and control sites were measured using the DRS technique. The amount of native pigment induced after the conditioning initial dose (300 J.cm^-2^) as well as after the subsequent 150 J.cm^-2^ doses for different skin types (Fig 6A). DRS measurements show similar skin response as clinical observations. The facultative pigment increased with multiple VL irradiations and this increase is more pronounced in the darker skin complexion. This could be as a result from increased melanogenic activity in darker skin types as compared to Caucasian skin. Also the type of melanin produced in melanosomes and the size, number and packaging of melanosomes, with melanin content of melanosomes ranging from 17.9% to 72.3% in Caucasian skin versus African American skin respectively plays a huge role in deciding the skin color in a given ethnicity [25, 26].

The absorption spectrum of the irradiated sites with VL subtracted from the control surrounded non-exposed area showed different spectral characteristics at each timepoint (Fig 6B). Spectral characteristic of VL induced pigment is different from that of native epidermal melanin. The spectrum at day 1, day 2 and 3 illustrate the spectral absorbance of the irradiated site immediately after irradiation with similar spectral characteristics of an IPD response. Fig 6B also shows the absorption spectrum of the irradiated site on day 10 and the absorption spectrum of melanin in skin. It could be speculated that the pigment formed at earlier time points is a mixture of products of photo-oxidation and or various precursors of melanin and various metabolites altogether whereas the pigment formed at later time points appears to be native pigment. Based on *ex-vivo* results the gene involved in melanogenesis gets turned on a later time points that results in de novo pigment formation.

## Discussion

VL comprises about 44% of solar radiation and is the only portion of the solar spectrum visible to the human eye. If we consider that the solar irradiance in the visible range is about 50 mW.cm^-2^, the dose used in this study (150 J.cm^-2^) would be equivalent to approximately 50 minutes in the sun light (time based unpublished data from N.K. obtained from Kuwait City, Kuwait which has same latitude of Tallahassee, FL). The data presented in this paper suggested one exposure with VL can produce transient pigmentation in Caucasian Skin but clinically persistent pigmentation is more evident with multiple exposures both under *in vivo* and *ex-vivo* conditions. Pre-exposure of skin which is termed as skin conditioning is required to induce pigmentation.

Skin plays a vital role as a physical barrier against external influences as well as act as a neuroendocrine organ, which helps to regulate global homeostasis. Skin cells produce hormones, neurotransmitters and neuropeptides and corresponding functional receptors in response to stressors including UV [8]. As a result both UVB and UVA bands of the solar spectrum have been shown to be melanogenic, resulting in different cutaneous pigmentary response in relation to respective exposures. UVA radiation induces an immediate pigment response. The mechanism of action for immediate UVA induced pigmentation is believed to be the result of photochemical alteration of melanin and of melanin precursors and metabolites followed by neo-melanogenesis [27–29], whereas with UVB exposure it is due to increase in the number of melanocytes, followed by increase in melanin production and transfer to keratinocytes [19]. At molecular level, increase in melanin content is directly correlated to increased release of alpha- melanocyte-stimulating hormone (alpha-MSH) in cutaneous keratinocytes and melanocytes when exposed to UVB resulting in increased melanogenesis [30]. In addition, UV induced increased nitric oxide (NO) is also considered as one of the melanogenesis that can play an important role in UV induced hyperpigmentation in vivo. NO-stimulated melanogenesis has been shown to work through activation of cyclic guanosine 3,5-monophosphate (cGMP) pathway [31, 32].

In the present study, we have shown the induction of pigmentation with VL both *in vivo* and *ex-vivo*. The mechanism of action for the production of immediate pigmentation following exposure with VL is believed to be photo-chemical in nature; however, the mechanism of action for VL induced delayed pigmentation may be a result of neo-melanogenesis.

Human skin explants from Caucasian subjects when exposed to 150 J.cm^-2^ of VL daily for 5 days showed increased and perceptible pigmentation at day 7. Quantification of melanin both from VL exposed and sham treated explants extracted at day 7 showed increased melanin in VL exposed explants as compared to sham treated explants (Fig 1). It can be hypothesized that increase in Tyrosinase activity on Day 3 resulted in increased oxidation of L-tyrosine to L-dopa that later on fuels in formation of melanin polymer. Further increase in tyrosinase enzyme activity throughout day 7 that corresponds very well with increased tyrosinase gene expression resulted in even higher oxidation of L-tyrosine, providing substrate for melanin polymer. It can be speculated that continued increased formation of L-dopa lead to melanin polymer formation by day 7 resulting in clinical perceptible pigmentation.

Contrary to these results, Mahmud BH. et al. [5] have shown no induction of pigmentation in Caucasian skin with experiments performed in the beginning of the winter (late December). That study used only one exposure with different doses, whereas in the present study we used 5 consecutive exposures with VL with dose of 150 J.cm^-2^ which is lower than the threshold dose necessary to produce persistent pigmentation. Similar to *in vivo* study; *ex-vivo* study showed that pre-exposure of explants to VL for a certain period of time is required to activate the melanogenesis process. This suggests that a conditioning dose with VL is needed to reach their threshold value, at which they can start the first steps in the neo-melanogenesis process.

In order to address the significant increase in pigmentation in VL exposed explants, the mechanism of action was explored both at genetic and protein level. Increased Tyrosinase activity has been considered as the main mechanism responsible for skin tanning, although other factors, such as the redistribution and the particle size of melanosomes have been shown to play critical role as well [33–35]. Increased pigmentation in *ex-vivo* model can be directly related to increased Tyrosinase enzyme activity in VL treated explants both at day 3 and 7, with higher activity at day 7 (Fig 3A and 3B) as compared to sham treatment. During the same time there was an increase in enzyme activity in sham treatments at day 7 as compared to day 3. This could result from oxidative stress produced as a result of culturing conditions [36]. Regardless of the increase of tyrosinase activity in sham group, there is a significant increase in tyrosinase activity in visible light treated explants. Apart from enzyme activity, this particular wavelength showed significant induction of Tyrosinase gene expression at day 7 with no change at day 3, which aligns very well with the further increase in Tyrosinase enzyme activity at day 7. Increased gene expression of respective gene also suggests that neo melanogenesis process was activated after multiple exposures with VL. It can be speculated that increased Tyrosinase activity at early time points have started building up the melanin polymers, whereas increase in Tyrosinase gene expression as well as even higher Tyrosinase activity at later time points would result in clinically visible pigmentation. Interestingly blue light (470 nm) has been recently explored from wound healing perspective and has been shown release NO from nitrosyl-hemoglobin or mitochondrial protein complexes. Based on the earlier findings between UV and NO, it can also be hypothesized that visible light induced NO can result in increased pigmentation in skin [37].

The *clinical* studies with exposure to VL confirm the *ex-vivo* studies. A pilot study performed in skin type V-VI during early December showed that the skin did pigment but the pigmentation did not persist and faded in the first hour after irradiation. Contrary to this Mahmoud BH. et al [5] showed that the threshold dose for PPD in subjects with Fitzpatrick skin types V and VI was 80–120 J.cm^-2^ and the induced pigmentation remain perceptible for at least 2 weeks. This result was intriguing and the only difference between the experiments presented in this paper and the ones by Mahmoud B.H. et al was that the later ones were conducted during the summer. The hypothesis here is that since subjects were more exposed to solar radiation during the summer than the winter time subjects have more epidermal pigmentation and/or precursors that are responsive to VL radiation. We considered the possibility that there is a need for conditioning with VL dose that can induce an initial pigmentation in skin and this initial pigmentation is the precursor for the subsequent pigmentation for the following exposures.

Skin pigmentation has been shown to have two distinct and independent absorption spectra, which depends on the type of UV radiation they have been exposed (UVB or UVA) Kollias N. et al [38] and Ouyang H. et al [39]. In this study we have shown that a single exposure to VL might prove ineffective in producing pigment. As observed, a second dose, one day later, can be as little as half of the first dose and prove more effective in producing lasting pigment in the skin and subsequent increase in skin darkening is observed with lower doses of VL. A third exposure—at half the original dose also results in a significant (visually) pigment that lasts. Exposure of the skin to what might be termed as “conditioning” dose has proved quite effective in augmenting the pigment produced (Fig 5). The initial melanogenic precursor, liable for VL, produced after irradiation (days 1 and 2) has spectral characteristics different from the native pigment (Fig 6B) but the pigment presented on day 10 looks very much like the native pigment. The spectral difference illustrates a continuous change from an UVA IPD induced type of skin reaction (day 1) to the formation of new melanin (day10).

Increase in melanin as a result to exposure to VL in skin can be suggested as response to stress induced by the respective light source. Skin has been recently categorized as an endocrine organ with its own HPA axis that gets activated by stress response [40]. In addition Leibel et al. [6] showed that skin produces ROS when exposed to visible light, which later results in increases matrix metalloproteinases resulting in accelerated photoaging. Increased melanin as a result of increased VL exposures can certainly be tagged to uneven pigmentation, which is one of the main components of photoaging and is a cosmetic concern. These findings do call out for sunscreens that could protection in visible light.

## Conclusion

In this manuscript the effect of multiple exposure of skin to VL were explored for pro-pigmentation activity. Interestingly multiple exposure with VL was able to induce pigmentation in explants extracted from Caucasian skin. Further exploration at biological endpoints suggested that besides pigment formation due to photo-oxidation activities, VL was able to activate the whole melanogenesis process. The clinical result of the current study confirm the *ex-vivo* studies and demonstrate that VL is able to induce pigmentation after multiple exposures, which suggests that preconditioning is required to activate the melanogenesis process. The findings also align very well with the findings from Mahmud et al., since both studies demonstrate that VL cannot produce persistent pigmentation with just one VL exposure, especially in subjects with Caucasian skin.

Taken together these results demonstrate that in addition to UV, VL can have significant impact on producing uneven pigmentation in skin which is a main factor in photoaging. Furthermore this is the first report that preconditioning of the skin with VL, followed by multiple exposures to VL, can result in pigment formation. Thus photoexposure and photodamage should not be considered strictly as a result of UV exposure since the skin is exposed to whole spectra of wavelengths including VL, and VL can induce photodamage pathways in a manner similar to UV.

## Materials and Methods

### Chemicals

Unless otherwise specified, all chemicals were purchased from Sigma-Aldrich (St Louis, MO, USA).

### Human skin explants and VL exposure

Human abdominal skin samples (otherwise to be discarded) were obtained following informed consent from healthy Caucasian females with age ranging from 38 to 59yrs undergoing abdominal plastic surgery (The Peer Group, Florham Park, NJ, USA). Patient identities were not disclosed to preserve confidentiality, in compliance with US HIPAA regulations. Punch biopsies (8 mm) were cultured in a 1:1 mixture of DMEM and F12 nutrient mixture (F-12) (Invitrogen, Carlsbad, CA) supplemented with 2% FBS (Invitrogen, Carlsbad, CA) and a cocktail of growth factors according to. Rossetti D et al.[41]. After an overnight incubation in a humidified chamber, in a 5% CO_2_ atmosphere at 32°C, explants were transferred to 37°C for the rest of the culture duration. Human skin explants were exposed to visible light 150 J.cm^-2^ (day 1, 2, 3, 6, 7) along with one set of sham treatments which were kept outside for the same time as visible light exposures but without any exposure to any light. One set of explants was harvested at day 3 and another set was harvested at day 7 both from visible light and sham treatments for gene expression analysis, enzyme activity assays and quantification of melanin content.

### Subjects

The clinical study was conducted in accordance to the Declaration of Helsinki principles. The clinical protocol was approved by the Allendale Institutional Review Board (Old Lyme, CT), and written informed consent was obtained from all subjects prior to enrollment. The enrolled subjects were in general good health and prescreened on the basis of Fitzpatrick skin type prior to enrollment.

### Light Source

The explants and subjects were irradiated with a modified Fiber-Lite Model 180 Illuminator (Dolan-Jenner Industries, Inc., Boxborough, MA). This light source consists of a 150 Watt Quartz halogen lamp that is capable of producing fluence rates in excess of 300 mW.cm^-2^ when filtered with three near infrared absorbing glass filters (KG5 3mm Schott (Duryea, PA) to attenuate IR radiation above 700 nm; and one glass filter to block wavelengths shorter than 400 nm (GG 400 3 mm Schott glass, Duryea, PA). The output of the light source was delivered with a glass optical fiber (10 mm diameter for the skin explants and 8 mm for the *in vivo* studies) and the fluence rate was measured with a calibrated Newport Multi Function Optical Meter Model 1835-C (Irvine, CA) with a VL sensor model 818-C. The visible light source used in this paper has exactly the same spectral irradiance as the one published in the Mahmoud’s paper [5]. The total amount of UVA (320–400 nm) in this light source is 0.19% over the total spectrum. This is achieved by using a series of cutoff filters and heat absorbing filters. For doses of 300 Jcm^-2^ of visible light used in the study for this work, the cumulative UVA dose of 0.6 Jcm^-2^, what would not be enough to induce a PPD in the tested subjects.

### Diffuse reflectance spectroscopy measurements

DRS is a non-invasive assessing tool to determine changes of chromophores in skin [42, 43] and we employed DRS for both *in vivo* and *ex-vivo* studies. The system consisted of a light source (Xe arc lamp Newport, Stratford, CT), a bifurcated optical fiber probe (Multimode Fiber Optics, Inc., NJ) and a spectrometer (PIXIS 256, Princeton Instruments, Trenton, NJ). The amount of pigment produced by the exposure was calculated by the area under the absorption spectrum at 620–720 nm, in which absorption change due to blood is relatively minimal. Measurements were performed four times per site for every time points.

### RNA extraction and QPCR

The expression level of Tyrosinase mRNA was quantified by QPCR. GAPDH was used as a house keeping gene and the relative abundance of mRNA obtained for both genes from respective samples was normalized to the GAPDH. The respective primers were purchased from Sabiosciences. Briefly RNA was extracted by using Trizol (Invitrogen). RNA concentration and purity was determined by measuring the absorbance using a NanoDrop (NanoDrop Technologies, Wilmington, DE, USA). Followed by reverse transcription using the Applied Biosystems RT-PCR kit and qPCR was performed using SYBR green on 7500 Realtime PCR system (Applied Biosystems, CA). Primers for respective gene expression assays were purchased from SA Biosciences.

### Melanin extraction from explants and Tyrosinase activity assay

Skim samples (1 g) were homogenized by sonication in 4 ml of RIPA buffer until the tissue was converted to liquid, then centrifuged at 1200 *g*. The liquid phase was stored at −80°C, which was later on used for measuring protein concentration and assessing tyrosinase enzyme acitivty. Pellets consisting of cell debris and black pigment were washed twice with PBS and were centrifuged again at 1200 *g*. The pellet was dissolved in 1 M NaOH to dissolve the pigment at 60°C and solubilized melanin was measured at 405nm.

Tyrosinase activity, as the dopa oxidase here, was assessed by determining the catalysis of L-DOPA to dopachrome, which has an absorption peak at 475 nm. The assay was performed in a total volume of 150 μl, including 50 μl of sample (equal to 30 μg of protein extract) and 100 μl of freshly prepared L-dopa solution (0.1% in PBS pH 6.8). The protein concentration of the enzyme was determined using BCA assay reagent. The assays were set up on ice in triplicate in 96-well round-bottom microtiter plates and were incubated for 4h at 37°C. The absorbance at 475 nm was measured with microplate reader (VersaMaxä tunable microplate reader, Molecular Devices, USA) to monitor the production of dopachrome, corrected for auto-oxidation of L-DOPA.

### Skin conditioning and multiple exposures

To assess the role of VL on skin, we performed multiple VL irradiation in a group of 14 subjects (5 males, 9 females; age range: 25–55; Fitzpatrick skin phototypes I-II, n = 5; II-III, n = 4, IV-V, n = 5). For in vivo studies dorsal forearm was used for exposure as it was suitable for experimentation purpose and the instruments could be easily used for the arms. Prior to irradiation subjects were allowed to rest for 15 minutes to acclimatize to the room temperature. The subjects were irradiated using the same light source used in the skin explants experiments.

Subjects at the day 1 were irradiated with an initial dose of 300 J.cm^-2^ with a fluence rate set at 150 mW.cm^-2^ in the dorsal forearm area. Digital photography and DRS measurements were performed on the irradiated site and an adjacently located un-exposed site used as control. The following day (day 2), prior to a second exposure, the previously treated area was imaged and DRS measurements were performed. A second exposure of 150 J.cm^-2^ (half of the initial dose) was delivered at the very same site with the same fluence rate. After irradiation the exposed site was imaged and the DRS reading taken from the exposed and control un-exposed site. On day 3 the same procedure as on day 2 was repeated. *In vivo* skin images were obtained with a Nikon D50 digital camera with a 60 mm Nikon lens coupled with a Canfield Twin Flash and close up set up (Canfield Scientific Inc, Fairfield, NJ). Cross polarized images and DRS were taken immediately after irradiation and at different time points.

### Statistical analysis

All exvivo data are presented as mean±SD. For exvivo data were analyzed using a Student’s t-test with significance set at p > 0.05. For in vivo study, data are expressed as the mean ± SD. Statistical significance was determined with unpaired two-tailed Student's t-test using the Minitab 15 software (Minitab Inc., State College, PA).

## Supporting Information

S1 Fig Proposed UV–VIS PPD Action Spectrum. The wavelength at 330, 350, 370 and 390 nm and the wavelength on the visible range 436 nm, 525 nm and 600 nm are estimated from the clinical trials.
(TIF)
