# Supplementary figures and images for: Visible Light Induces Melanogenesis in Human Skin through a Photoadaptive Response
Source: PLoS One. 2015 Jun 29;10(6):e0130949. doi: 10.1371/journal.pone.0130949 (PMC4488093; doi:10.1371/journal.pone.0130949)

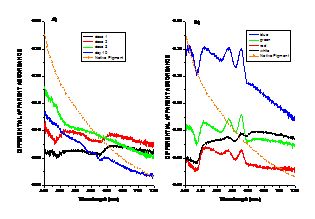

Supplement: S1 Fig — The wavelength at 330, 350, 370 and 390 nm and the wavelength on the visible range 436 nm, 525 nm and 600 nm are estimated from the clinical trials. (TIF) [file pone.0130949.s001.tif]
